# Gouty Tophus in the Scrotum: An Unusual Extra-Articular Manifestation of Gout

**DOI:** 10.31486/toj.20.0143

**Published:** 2021

**Authors:** Fred Alain Montelongo-Rodríguez, Pedro Antonio Madero-Morales, Adrián Mauricio Martínez-Fernández, Maria Alejandra Rodríguez-Abarca, Max Molina-Ayala, Adrián Gutiérrez-González

**Affiliations:** ^1^ Department of Urology, Hospital Universitario Dr. José Eleuterio González, Universidad Autónoma de Nuevo León, Monterrey, Nuevo León, Mexico; ^2^ Faculty of Medicine, Hospital Universitario Dr. José Eleuterio González, Universidad Autónoma de Nuevo León, Monterrey, Nuevo León, Mexico; ^3^ Department of Pathology, Hospital Universitario Dr. José Eleuterio González, Universidad Autónoma de Nuevo León, Monterrey, Nuevo León, Mexico

**Keywords:** *Gout*, *scrotum*, *tophus*, *uric acid*

## Abstract

**Background:** Gout is a chronic disorder caused by the deposition of monosodium urate crystals in soft tissues. Tophi are granulomatous inflammatory responses to the deposited crystals and manifest as subcutaneous nodules, typically in the first metatarsophalangeal joint but also in the olecranon bursa, Achilles tendon, ears, and finger pulps.

**Case Report:** A 56-year-old male presented to an outpatient clinic with an 8-month history of an expanding scrotal lesion. The patient had no significant family history but had a history of high blood pressure and gout, diagnosed at age 24 years, without current treatment. Excisional biopsy from the ulcerated area of the scrotum was performed for confirmatory diagnosis, and pathology reported gouty tophus.

**Conclusion:** To our knowledge, this case is the first report of a scrotal manifestation of gouty tophus and the second of genital involvement. Awareness of the possibility of genital manifestations of this disease is important because although gouty tophi are rare, they can present in patients with long-term uncontrolled gout.

## INTRODUCTION

Gout is a chronic disorder caused by monosodium urate (MSU) crystal deposition in joints and soft tissues.^1^ Gout is the most common inflammatory arthropathy, having an increased prevalence and incidence in developed countries, especially in North America and Europe.^2^ The clinical spectrum of the disease ranges from asymptomatic hyperuricemia to acute monoarthritis to tophus formation.^1^

Tophi are chronic granulomatous inflammatory responses to deposited crystals that manifest as subcutaneous nodules, typically at the first metatarsophalangeal joint.^3^ Tophi occur approximately 10 years after gout onset and represent the fourth stage of the disease.^3^ Gouty tophi occur in <10% of patients because of available treatment modalities.^4^

We report the case of a 56-year-old male with a medical history of gout and high blood pressure who presented with a large, indurated lesion on the scrotum.

## CASE REPORT

**Figure 1.**
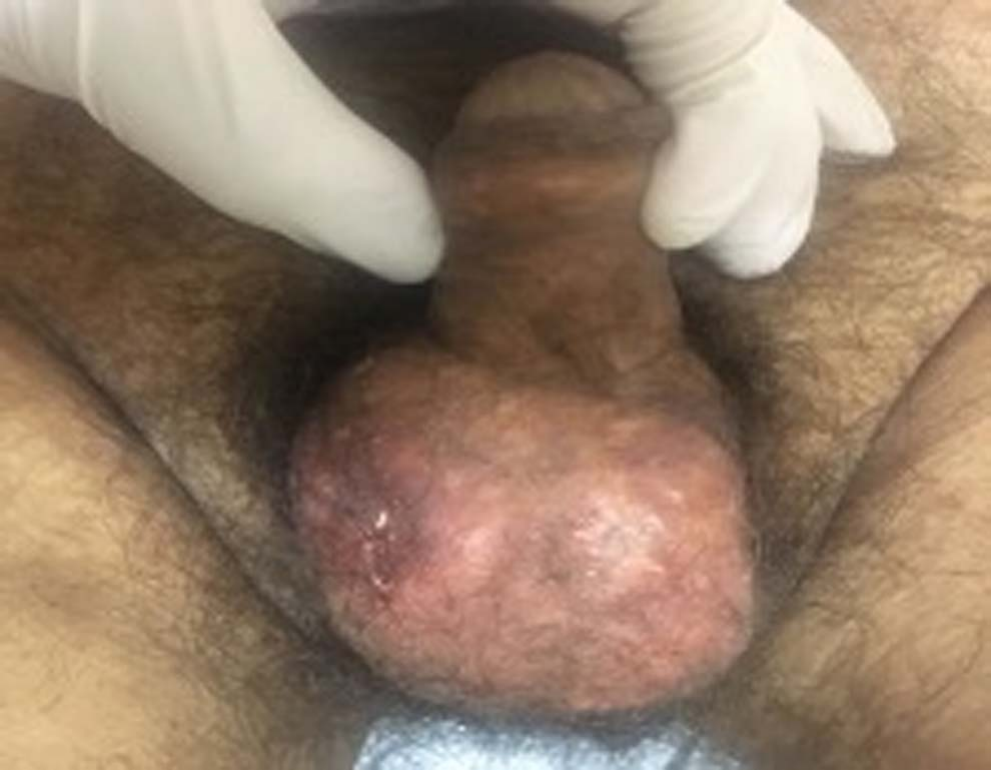
Tophus in the scrotum and plaque that covers the entire scrotum.

A 56-year-old male presented to an outpatient clinic with an 8-month history of an expanding, painless scrotal lesion. The patient reported that the lesion had started to secrete a whitish, odorless, thick fluid from a scrotal pustule 1 month prior to presentation. The patient had a history of high blood pressure and gout, diagnosed at age 24 years. He did not receive any specific treatment for either disease. Findings during the physical examination were blood pressure of 160/100 mmHg; multiple nodules at the elbows, helix, forearms, and hands; and a deformity in the right knee that contributed to a movement limitation. Genital exploration revealed a scrotal plaque that embraced the totality of the scrotum, measuring approximately 5 × 8 × 1.5 cm, with a right hemiscrotal pustule and a white, granular, inodorous discharge (Figure 1). Both testicles were inside the scrotum and were soft in consistency but difficult to palpate.

**Figure 2.**
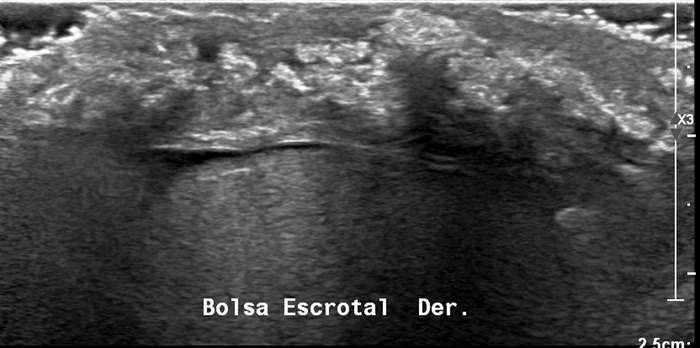
Ultrasound shows an increase in scrotal thickness; areas with increased echogenicity correspond to calcified nodules, some of which project posterior acoustic shadow corresponding to a gouty tophus.

**Figure 3.**
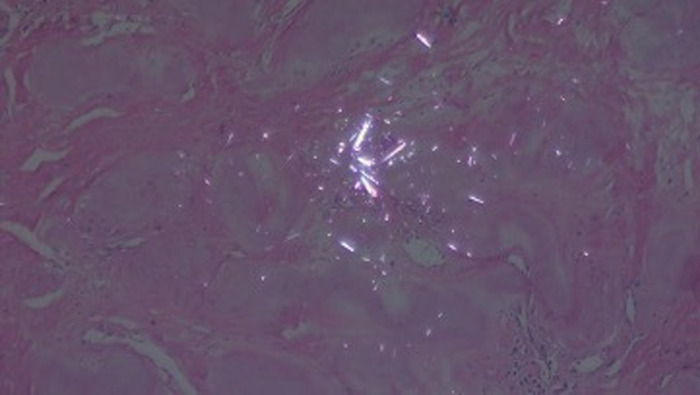
Scrotum skin biopsy shows abundant eosinophilic and amorphous cellular material at the level of the deep dermis, as well as an inflammatory infiltrate of chronic predominance. Although the biopsy was processed, a few monosodium urate crystals could still be observed under polarized light.

Laboratory workup revealed normocytic normochromic anemia, hemoglobin of 11.7 g/dL (reference range, 13.8-17.2 g/dL); uric acid of 11.4 mg/dL (reference range, 3.4-7.0 mg/dL); creatinine of 2.1 mg/dL (reference range, 0.6-1.1 mg/dL); calculated glomerular filtration rate of 34 mL/min/1.73 m^2^ (reference range, >90 mL/min/1.73 m^2^); and a rheumatoid factor of 19 IU/mL (reference, 20 IU/mL). Scrotal ultrasound revealed multiple soft tissue scrotal calcified nodules (Figure 2). Renal ultrasound showed evidence of chronic kidney disease without evidence of lithiasis. Pathology report of an excisional biopsy from the ulcerated area of the scrotum was gouty tophus (Figure 3).

The patient was referred to the rheumatology department, where lifestyle changes such as low alcohol intake and a low-protein diet were suggested, and medical management was initiated, with colchicine 0.5 mg every 3 days for 6 months and allopurinol 100 mg daily for 6 months. Surveillance of the lesion continued for the first 6 months of medical treatment, with complete healing of the ulcerated area but poor improvement of the scrotal plaque. Removal of the scrotal plaque was proposed, but the patient denied this surgical procedure and stopped attending subsequent appointments to assess the response to the medical treatment the rheumatologist offered.

## DISCUSSION

To our knowledge, this case is the first report of a scrotal manifestation of gouty tophus and the second report of genital involvement. A report of penile tophi is the other case of genital involvement.^5^ Our patient had poor adherence to medical treatment, which made it impossible to follow his scrotal injury and gout.

Classic MSU crystal deposition sites are the first metatarsophalangeal joint, olecranon bursa, Achilles tendon, ears, and finger pulps,^3,6^ consistent—other than the Achilles tendon—with our patient's nodules. Several extra-articular presentations of tophus have been reported, including bronchi, mitral valve, liver, and breast tophi,^7-10^ but genitourinary involvement is rare.

The gold standard for the diagnosis of gout is the identification of MSU crystals in synovial fluid or tophus aspirate.^11^ In our case, we took a sample of the scrotal injury and found crystals in the microscopy, giving us the diagnosis of the scrotal lesion without needing to sample the synovial fluid.

Ultrasound is an inexpensive and noninvasive test for tophi diagnosis.^12^ Characteristic ultrasonographic extra-articular tophus lesion findings include a circumscribed, inhomogeneous, hyperechoic, and/or hypoechoic aggregation that may or may not generate posterior acoustic shadow,^13^ consistent with our patient's ultrasound findings.

Treatment of chronic tophaceous gout consists of 2 modalities: (1) lifestyle management with dietary changes, low alcohol intake, and good blood pressure control to help keep uric acid levels down and (2) pharmacotherapy.^14^ First-line medications for urate-lowering therapy include xanthine oxidase inhibitors, such as allopurinol and febuxostat. Second-line urate-lowering therapies include uricosuric drugs, such as probenecid and benzbromarone.^11^ Newer agents include recombinant uricase, which is a third-line treatment for uncontrollable gout but shows the best long-term results for tophi dissolution.^14^

In our case, the rheumatologist prescribed colchicine and allopurinol because pegloticase, a pegylated recombinant uricase that catalyzes the oxidation of uric acid into allantoin, is not readily available at our center.^14^ Allopurinol was given as prophylaxis against flares during the urate-lowering therapy.^14^ Tophi can reduce in size with treatment, but complete response is rare. No published guidelines state how to treat this pathologic finding; however, as Flores Martín et al mention in their study, surgical treatment can be proposed for patients with no positive response to medical treatment,^5^ but our patient refused surgical management.

## CONCLUSION

Unusual extra-articular manifestations of gouty arthritis can respond positively to proper treatment and management. Multidisciplinary management, including rheumatologists, cardiologists, nephrologists, and nutritionists, is the best treatment modality. In our case, we were the first specialists to have contact with the patient in our institution, which for our daily medical practice is uncommon. Although no published guidelines discuss management of scrotal manifestations of gouty tophus, urologists need to know that these lesions can occur in patients with gout.

## Notes

*This article meets the Accreditation Council for Graduate Medical Education and the American Board of Medical Specialties Maintenance of Certification competencies for Patient Care and Medical Knowledge.*
